# Does the incorporation of strontium into calcium phosphate improve bone repair? A meta-analysis

**DOI:** 10.1186/s12903-022-02092-7

**Published:** 2022-03-08

**Authors:** Ming-Dong Yan, Yan-Jing Ou, Yan-Jun Lin, Rui-Min Liu, Yan Fang, Wei-Liang Wu, Lin Zhou, Xiu Yao, Jiang Chen

**Affiliations:** 1 Fujian Key Laboratory of Oral Diseases and Fujian Provincial Engineering Research Center of Oral Biomaterial and Stomatological Key Laboratory of Fujian College and University, School and Hospital of Stomatology, Fujian Medical University, Fuzhou, 350002 China; 2 Department of Oral Implantology, Affiliated Stomatological Hospital of Fujian Medical University, Fuzhou, 350002 China; 3 ORAL Center, Fujian Provincial Governmental Hospital (Affiliated Hospital of Fujian Health College), Fuzhou, 350003 China; 4 Fujian Provincial Key Laboratory of Polymer Materials, College of Chemistry and Materials Science, Fujian Normal University, Fuzhou, 350007 China; 5 Institute of Stomatology and Research Center of Dental and Craniofacial Implants, School and Hospital of Stomatology, Fujian Medical University, Fuzhou, China

**Keywords:** Bone defects, Calcium phosphate, Strontium, Bone regeneration, Meta-analysis

## Abstract

**Background:**

The application of calcium phosphate (CaP)-based bone substitutes plays an important role in periodontal regeneration, implant dentistry and alveolar bone reconstruction. The incorporation of strontium (Sr) into CaP-based bone substitutes appears to improve their biological properties, but the reported in vivo bone repair performance is inconsistent among studies. Herein, we conducted a systematic review and meta-analysis to investigate the in vivo performance of Sr-doped materials.

**Methods:**

We searched PubMed, EMBASE (via OVIDSP), and reference lists to identify relevant animal studies. The search, study selection, and data extraction were performed independently by two investigators. Meta-analyses and sub-group analyses were conducted using Revman version 5.4.1. The heterogeneity between studies were assessed by I^2^. Publication bias was investigated through a funnel plot.

**Results:**

Thirty-five studies were finally enrolled, of which 16 articles that reported on new bone formation (NBF) were included in the meta-analysis, covering 31 comparisons and 445 defects. The overall effect for NBF was 2.25 (95% CI 1.61–2.90, p < 0.00001, I^2^ = 80%). Eight comparisons from 6 studies reported the outcomes of bone volume/tissue volume (BV/TV), with an overall effect of 1.42 (95% CI 0.65–2.18, p = 0.0003, I^2^ = 75%). Fourteen comparisons reported on the material remaining (RM), with the overall effect being -2.26 (95% CI − 4.02 to − 0.50, p = 0.0009, I^2^ = 86%).

**Conclusions:**

Our study revealed that Sr-doped calcium phosphate bone substitutes improved in vivo performance of bone repair. However, more studies are also recommended to further verify this conclusion.

**Supplementary Information:**

The online version contains supplementary material available at 10.1186/s12903-022-02092-7.

## Introduction

At present, the repair of alveolar bone defects caused by disease, trauma, periodontitis, or congenital malformation is facing challenge, especially for patients with large bone defects or systemic diseases (such as osteoporosis or diabetes) [1, 2]. Although autologous bone grafting is considered to be the gold standard, its clinical applicability is limited owing to the need to open up a second surgical area and possible complications at the donor site [3, 4].

Nowadays, calcium phosphate (CaP) ceramics—as a representative material for synthetic bone substitutes—have been widely used in periodontal regeneration and alveolar bone reconstruction [4, 5]. However, traditional calcium phosphate materials have insufficient osteogenic ability and degradation performance. To improve the biological properties of these bone substitutes, researchers have attempted to incorporate bioinorganic ions into CaP–based materials [6].

Among various bioinorganic ions, strontium (Sr) has attracted significant research attention in the past ten years [7]. Sr is known to be a trace element in the human body and plays an important role in bone metabolism [8, 9]. It is conducive to osteogenesis, and can be mixed with hydroxyapatite (HA) through surface exchange or ion substitution, leading to the increase of bone mineral content and bone density, which improves bone regeneration and repair.

Several studies have investigated the effects of the addition of Sr on the physicochemical properties and in vitro/in vivo behaviour of CaP-based bone substitutes. Tao et al. demonstrated that the calcium phosphate doped with Sr has a faster absorption rate [10]. In addition, Sr-substituted biomaterials increased the differentiation of osteoblasts and activated the expression of pro-osteogenic molecules used for bone remodelling [11–13]. A number of in vivo studies have shown that Sr-enhanced calcium phosphate materials have better osteogenic properties in vivo [14–16]. However, other study found that no positive effect was observed in terms of promoting in vivo bone regeneration [17].

In view of the differences among studies regarding the effects of Sr-doped CaP-based materials, it becomes imperative to conduct a systematic review and meta-analysis. In addition, sub-group analyses based on different animals, material types, and implantation periods were also conducted. The main purpose of this study was to systematically review the synthesis method and characteristics—such as crystallinity, particle size, and porosity—of included Sr-doped (CaP) materials and to analyse the properties of new bone formation (NBF) and material degradation in vivo.

## Methods

### Search strategy

The methodology of this study followed the recommendations of the Systematic Review Centre for Laboratory Animal Experimentation (SYRCLE) guidelines [18] and the guidelines of the PRISMA statement (http://www.prisma-statement.org/). In vivo studies that evaluate the effects of Sr-doped (CaP)-based materials from database inception to December 2020, without any language restrictions, were identified by searching the PubMed and EMBASE (via OVIDSP) databases. This paper combined the MeSH heading and text search strategies, with multiple terms associated with ‘bone regeneration’, ‘strontium’, ‘bone substitutes’, and ‘animal research’ were used. Since tricalcium phosphate (TCP), HA, anhydrous dicalcium phosphate (TTCP), and tetracalcium phosphate (DCPA) are commonly used materials in this field of research, these terms have been also used as search words in the search formula. Search filters were utilized to detect all publications concerning animal studies [19, 20]. The detailed search strategies for PubMed and Embase are shown in Additional file 1: Table S1 and Additional file 2: Table S2, respectively. In addition, we manually searched the reference lists of major research journals and review papers to identify additional relevant studies.

### Eligibility criteria

Two investigators (Y-M.D. and L-R.M.) independently screened potentially eligible studies. Any disagreement was resolved by discussion and consensus among reviewing authors. The inclusion criteria were (1) original animal studies on bone defects, (2) comparisons of Sr-doped and Sr-free (CaP)-based bone substitutes; and (3) outcomes of histological, micro-CT, or histomorphometric data.

### Study selection and data extraction

Two authors independently reviewed studies considered for inclusion in the meta-analysis and performed data extraction. We used an existing data extraction method to retrieve data regarding the basic characteristics. For all included papers, the outcome data for the experimental and control groups were extracted if the mean, standard deviation (SD) or standard error (SE), and the number of defects per group (N) were reported or could be recalculated. If the data were presented only in graphical form, pictures were converted to data using the WebPlotDigitizer tool (available online at https://automeris.io/WebPlotDigitizer/), which was considered to have high levels of intercoder reliability and validity [21].

### Quality assessment

The risk-of-bias assessment was based on SYRCLE’s Risk of Bias (RoB) tool, which is specifically designed for animal studies. Two authors independently assessed the risk of bias.

### Statistical analysis

The primary outcome of interest was the pooled overall NBF. Bone volume/tissue volume (BV/TV) and remaining material (RM) were the second outcomes of interest. Quantitative meta-analysis was performed when more than one study presented relevant data. Standardized mean differences (SMD) or mean differences (MD) and 95% confidence interval (95% CI) were calculated. Heterogeneity was assessed using I^2^. An I^2^ value greater than 50% was considered to indicate significant heterogeneity. However, because of the underlying methodological heterogeneity (e.g. baseline characteristics of the animals, sample sizes, and implantation periods), we used the DerSimonian and Laird random-effects model for all analyses. Potential sources of between-study heterogeneity were explored by subgroup analyses according to the following factors, whenever appropriate: physical condition (health vs disease); animals (e.g. rat, rabbit, or sheep); materials; and implantation periods. We reported p-values for each covariate. Publication bias was investigated through a funnel plot. Analyses were conducted using Review Manager (version 5.4.1, The Cochrane Collaboration, 2020).

## Results

### Paper identification and selection

**Fig. 1:**
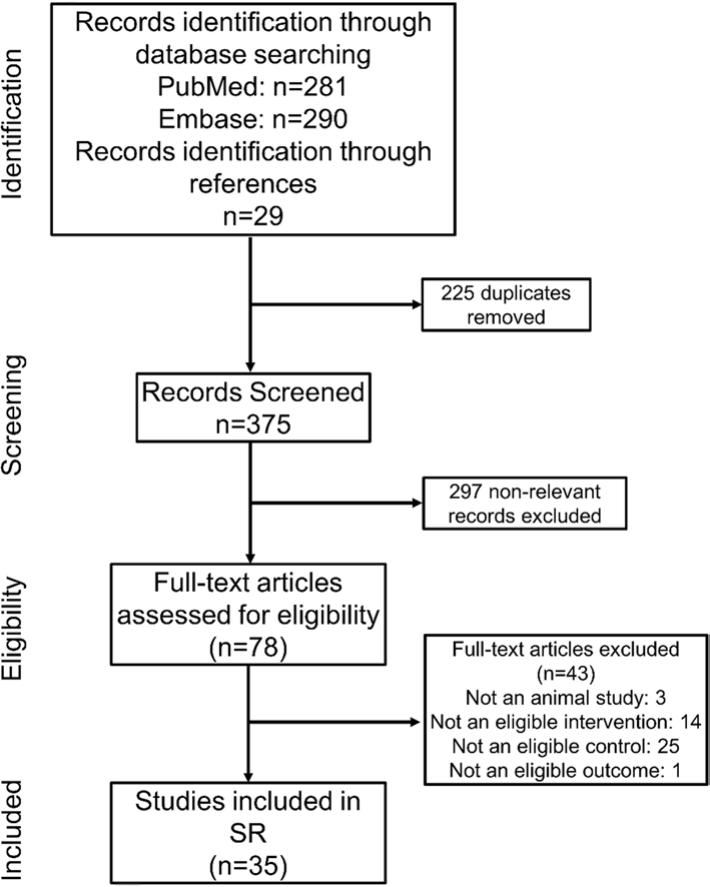
Search flowchart

Through the search, a total of 600 related articles were retrieved, including 281 from Pubmed, 290 from Embase, and 29 from reference lists. After removing duplicates and screening all titles and abstracts, 78 potential studies were selected for full-text evaluation. Finally, 35 papers [10, 11, 13–17, 22–49] met the inclusion criteria and were included in the systematic review (Fig. 1).

### Characteristics of included studies

**Fig. 2:**
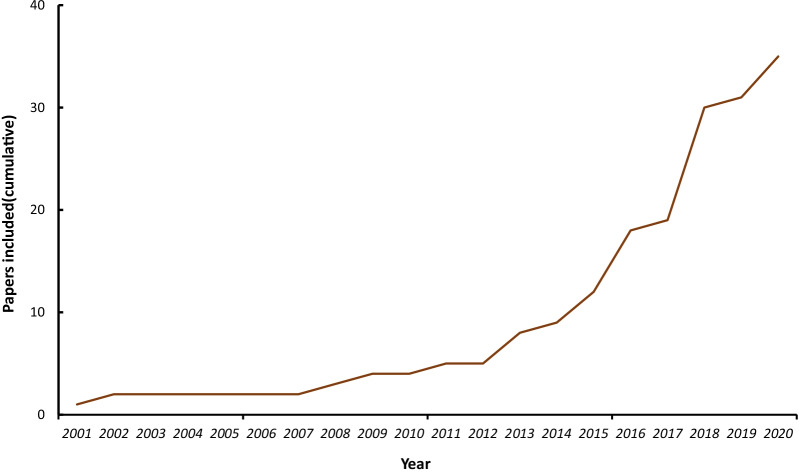
Cumulative number of papers included in the systematic review each year

The 35 included studies were published from 2001 to 2020, and the curve of the cumulative number of papers included in the systematic review each year is shown in Fig. 2, indicating the increasing amount of attention this topic has received in the past decade.

Among these studies, one used a dog model [48], one used a mouse model [46], three used sheep models [26, 39, 41], thirteen used rabbit models [11, 13, 15, 28, 30, 31, 33–35, 37, 38, 45, 47], and seventeen used rat models [10, 14, 16, 17, 22–25, 27, 29, 32, 36, 40, 42–44, 49]. There were nineteen studies focused on healthy animals [15, 23, 28–31, 33, 35–41, 45–49], one on albinism [24], one on osteonecrosis [34], and thirteen on osteoporosis [10, 11, 14, 16, 17, 22, 25–27, 32, 42–44]. The main characteristics of the included studies are listed in Table 1.

**Table 1:** Characteristic of papers included

References	Animals/surgical site	Biomaterials	Strontium	Number of defects per group and per time point	Implantation period	New bone formation (mean ± SD)	Remaining material (mean ± SD)	Conclusion of bone repair	Conclusion of biomaterial resorption
Baier [22]	Rat/Osteoporosis/Femur	CPC^a^/SPC	NA	10	1,3 and 6 months	Histomorphometrical results 1 m: 0 (CPC) 0.157 ± 0.096 (SPC) 3 m: 0.331 ± 0.163 (CPC) 0.398 ± 0.247 (SPC) 6 m: 0.325 ± 0.193 (CPC) 3.789 ± 0.687 (SPC)	NA	Sr-doping improved bone repair	NA
Carmo [23]	Rat/Healthy/Maxilla	CHA/SrCHA	NA	5	1 and 6 weeks	Histomorphometrical results 1w: 18.2 ± 2.04(CHA) 17 ± 1.7(SrCHA) 6w: 28.2 ± 3.82(CHA) 32 ± 4.15(SrCHA)	Histomorphometrical results 1w:14.6 ± 2.50(CHA) 18.9 ± 1.69(SrCHA) 6w: 16.5 ± 2.41(CHA) 10.4 ± 2.33(SrCHA)	Sr-doping did not improve bone repair	Sr-doping did not reduce biomaterial resorption
Cassino [24]	Rat/Albinoism/Tibia	HA/HASr/HAGa	NA	10	7 days	NA	NA	NA	NA
Chandran [25]	Rat/Osteoporosis/Femur	Sham/HA/SrHA	10	6	8 weeks	Histomorphometrical results 0.68 ± 0.08(HA) 0.92 ± 0.04(SrHA)	NA	Sr-doping improved bone repair	NA
Chandran [26]	Sheep/Osteoporosis/Femur	SrHA—Ca9Sr1(PO4)6(OH)2 /HA–Ca10(PO4)6(OH)2 cHA/cSrHA	10	2	2 months	Histomorphometrical results 0.08 ± 0.03(HA) 0.24 ± 0.04(SrHA) 0.30 ± 0.06(cHA) 0.38 ± 0.04(cSrHA)	NA	Sr-doping improved bone repair	NA
Cheng [27]	Rat/Osteoporosis/Femur	Sham/CPC^a^/Sr-CPC	NA	7	6 weeks	NA	NA	NA	NA
Dagang [28]	Rabbit/Healthy/Femur	HAC/Sr-HAC	5t%, 10%	1	4, 8, 12 and 24 weeks	NA	NA	NA	NA
Elgali [29]	Rat/Healthy/Femur	DBB/HA/SrHA/Sham	50%	6	12 h, 3 and 6 days	Histomorphometrical results 6d: 4.575 ± 1.018(HA) 7.401 ± 0.635(SrHA)	NA	Sr-doping improved bone repair	NA
Ge [13]	Rabbit/NA/Femur	PLLA/HA/PLLA/Sr-HA/PLLA	NA	NA	5 weeks	NA	NA	Sr-doping improved bone repair	NA
Gu [30]	Rabbit/Healthy/Mandible	HA/Sr-HA	5t%, 10%	4	1, 3 and 6 months	NA	NA	NA	NA
Gu [31]	Rabbit/Healthy/Radius	HA/CPP/SCPP	NA	4	4, 8 and 16 weeks	NA	NA	Sr-doping improved bone repair	NA
Guo [32]	Rat/Osteoporosis/Femur	nano-HA/SrR nano-HA	200 mM	10	4 and 8 weeks	Histomorphometrical results 4w: 4.632 ± 1.105(nano-HA) 6.533 ± 0.812(SrRnano-HA) 8w: 7.752 ± 0.39(nano-HA) 9.832 ± 0.585(SrRnano-HA) Micro-CT 4w: 48.934 ± 0.842(nano-HA) 49.871 ± 3.556(SrRnano-HA) 8w: 51.930 ± 1.871(nano-HA) 58.573 ± 4.866(SrRnano-HA)	NA	Sr-doping improved bone repair	NA
Hu [33]	Rabbit/Healthy/Radius	Blank control/HA/Sr-HA	5 wt%	10	8 and 12 weeks	NA	NA	NA	NA
Kang [34]	Rabbit/Osteonecrosis/Femur	Autogenous cancellous bone/SrCPP/CPP	NA	4 (4 and 8 weeks) 8 (12 weeks)	4, 8 and 12 weeks	NA	NA	Sr-doping improved bone repair	Sr-doping reduced biomaterial resorption
Kaygili [35]	Rabbit/Healthy/Tibia	HA/SrHA	0.45, 0.9, 1.35, 1.8, 2.25 at.%	28	4 weeks	NA	NA	Sr-doping improved bone repair	NA
Kuang [36]	Rat/Healthy/Femur	CPC^b^/Sr-CPC	5t%, 10%	5	32 weeks	NA	NA	Sr-doping improved bone repair	Sr-doping accelerated biomaterial resorption
Li [14]	Rat/Osteoporosis/Femur	HA/SrHA	10 mol%	13	12 weeks	Histomorphometrical results 35.753 ± 1.815(HA) 53.721 ± 10.98(SrHA)	NA	Sr-doping improved bone repair	NA
Liao [37]	Rabbit/Healthy/Mandible	Blank/HA/Sr-HA	5t%, 10%	4	1, 3 and 6 months	NA	NA	NA	NA
Luo [38]	Rabbit/Healthy/Calvaria	HA/Sr-HA	NA	NA	4, 8 and 12 weeks	micro-CT: 12w:11.05 ± 1.11%(HA) 15.95 ± 3.23%(Sr-HA)	NA	Sr-doping improved bone repair	NA
Machado [39]	Sheep/Healthy/Tibia	Blood clots/HA/SrHA	1% (w/w)	5	30 days	Histomorphometrical results 31.2 ± 14.7%(HA) 26.2 ± 12.1%(SrHA)	Histomorphometrical results 36.2 ± 8.5%(HA) 51.2 ± 14.1% (SrHA)	Sr-doping did not improve bone repair	Sr-doping reduced biomaterial resorption
Masaeli [40]	Rat/Healthy/Calvaria	Control/CPC^c^/SrCPC	3 wt%	10	4 weeks	NA	NA	NA	NA
Reitmaier [41]	Sheep/Healthy/Femur and tibia	CPC^a^/SrCPC	NA	7	6 and 26 weeks	Histomorphometrical results 6w: Unoaded: 9.205 ± 2.092(CPC) 11.297 ± 5.021(SrCPC) Loaded: 11.715 ± 3.766(CPC) 13.389 ± 5.439(SrCPC) 26w: Unoaded: 13.158 ± 4.699(CPC) 29.323 ± 18.045(SrCPC) Loaded: 25.0 ± 5.827(CPC) 44.173 ± 4.511(SrCPC)	Histomorphometrical results 6w: Unoaded: 58.779 ± 12.023(CPC) 41.984 ± 22.138(SrCPC) Loaded: 59.160 ± 12.977(CPC) 43.702 ± 17.176(SrCPC) 26w: Unoaded: 63.254 ± 10.42(CPC) 36.746 ± 16.271(SrCPC) Loaded: 59.415 ± 10.055(CPC) 41.133 ± 10.786(SrCPC)	Sr-doping improved bone repair	Sr-doping accelerated biomaterial resorption
Salamanna [42]	Rat/Osteoporosis/Vertebra	HA/SrHA/HA-AL7/HA-AL28	3.1 atom%,6.9 atom%	10	8 weeks	NA	NA	Sr-doping improved bone repair in osteoporotic bone	NA
Tao [10]	Rat/Osteoporosis/Femur	Control/CPC^b^/SrCPC/BSrCPC	SrCO_3_: 5 wt%	5	8 weeks	Histomorphometrical results 22.222 ± 2.963(CPC) 33.333 ± 3.704(SCPC) Micro-CT: 0.345 ± 0.084(CPC) 0.4 ± 0.084(SCPC)	Histomorphometrical results 52.222 ± 7.037(CPC) 40.185 ± 5.741(SCPC)	Sr-doping improved bone repair	Sr-doping accelerated biomaterial resorption
Tao [43]	Rat/Osteoporosis/Femur	Control/β-TCP/Srβ-TCP/Asp-Sr β-TCP	10 wt%	10	8w	Histological results 30.573 ± 2.548(β-TCP) 45.223 ± 5.095(Sr /β-TCP) Micro-CT: 26.222 ± 2.667(β-TCP) 37.333 ± 3.556(Sr /β-TCP)	Histomorphometrical results 29.968 ± 3.048(β-TCP) 25.016 ± 2.413(Sr /β-TCP)	Sr-doping improved bone repair	Sr-doping accelerated biomaterial resorption
Thormann [44]	Rat/Osteoporosis/Femur/n = 15	Sham/CPC^a^/SrCPC	NA	15	6 weeks	Histomorphometrical results 4.2 ± 3(CPC) 11 ± 1(SrCPC)	NA	Sr-doping improved bone repair	NA
Tian [45]	Rabbit/Healthy/Radius	CPP/SrCPP	NA	8	4, 8 and 16 weeks	Histomorphometrical results 4w: 9.884 ± 0.401(CPP) 13.968 ± 0.560(SCPP) 8w: 19.012 ± 0.801(CPP) 27.179 ± 1.121(SCPP) 16w: 39.911 ± 1.121(CPP) 45.036 ± 1.361(SCPP)	4w: 25.054 ± 1.125(CPP) 25.696 ± 0.858(SCPP) 8w: 20.125 ± 0.857(CPP) 19.964 ± 0.482(SCPP) 16w: 12.411 ± 0.643(CPP) 11.875 ± 0.75(SCPP)	Sr-doping improved bone repair	Sr-doping accelerated biomaterial resorption
Tohidnezhad [46]	Mouse/Healthy/Femur	Sham/β-TCP/Srβ-TCP	NA	NA	2 months	Histological results 26.41% ± 1.31%(β-TCP) 61.93% ± 3.04%(β-TCP + Sr)	NA	Sr-doping improved bone repair	NA
Valiense [47]	Rabbit/Healthy/Maxilla	CHA/SrCHA	NA	6	4 and 12 weeks	Histomorphometrical results 4w: 17.812 ± 9.423(CHA) 16.890 ± 9.797(SrCHA) 12w: 27.964 ± 4.863(CHA) 31.368 ± 2.614(SrCHA)	Histomorphometrical results 4w: 14.620 ± 5.186(CHA) 18.241 ± 9.389(SCHA) 12w: 17.168 ± 7.869(CHA) 10.317 ± 6.36(SCHA)	NA	Sr-doping accelerated biomaterial resorption
Vestermark [48]	Dog/Healthy/Humerus	HA/SrHA/Allograft	NA	10	4 weeks	Histomorphometrical results 28 ± 5.1(HA) 36 ± 3.06(SrHA)	NA	NA	NA
Xie [15]	Rabbit/Healthy/Calvaria	CPP/SrCPP	NA	3	4, 8 and 12 weeks	Histomorphometrical results 8w: 18.938 ± 0.486(CPP) 25.475 ± 0.56(SCPP) 16w: 26.745 ± 1.344(CPP) 36.307 ± 0.198(SCPP)	NA	Sr-doping improved bone repair	NA
Yu [49]	Rat/Healthy/Calvaria	Coll scaffold/(APMs/coll scaffold)/(SrAPMs/coll Scaffold)	10 mol%	12	8 weeks	Micro-CT-BV/TV- 20.64 ± 7.33%(APMs/coll) 48.30 ± 11.75%(SrAPMs/coll)	NA	Sr-doping improved bone repair	NA
Yuan [17]	Rat/Osteoporosis/Femur	HA/SrHA/(HA/G3-K PS)/(SrHA/G3-K PS)	15%	6	8 weeks	Micro-CT: 17.558 ± 3.786(HA) 18.491 ± 3.567(15SrHA)	NA		NA
Zarins [11]	Rabbit/Osteoporosis/Femur	Sham/(HA /TCP)/Sr + (HA/TCP)	NA	7	12 weeks	NA	NA	Sr-doping improved bone repair	NA
Zhao [16]	Rat/Osteoporosis/Femur	WCP/SrWCP/Sr-Ran + WCP	NA	12	1, 8 and 12 weeks	Histological results 8w:10.267 ± 3.850(WCP) 19.037 ± 4.92(SrWCP) 16w:11.337 ± 3.422(WCP) 23.102 ± 3.422(SrWCP) Micro-CT: 8w:20.315 ± 0.945(WCP) 21.417 ± 2.205(SrWCP) 16w:21.889 ± 2.205(WCP) 26.457 ± 1.889(SrWCP)	NA	Sr-doping improved bone repair	NA

CPC: calcium phosphate cement; SPC: Sr-doping calcium phosphate cement; HA: hydroxyapatite; CHA: Carbonated hydroxyapatite; CPP: calcium polyphosphate; TCP: tricalcium phosphate; Sr: Strontium; HAC: hydroxyapatite cement; DBB: Deproteinized bovine bone; Sham: without graft materials; PLLA: poly( l—lactic acid); SCPP: strontium-doped calcium polyphosphate; Asp-Sr/β-TCP: strontium-doped β-tricalcium phosphate (Sr/β-TCP) modified with aspirin; BSrCPC: strontium-doped calcium phosphate cement combined with single-dose local administration of BMP-2;Coll: collagen; APMs: amorphous calcium phosphate porous microspheres; HA/G3-K PS: hydroxyapatite gel modified by integrating branched poly(epsilon-lysine) dendrons with third-generation branches exposing phosphoserine; WCP: hydroxyapatite whiskers; Sr-Ran: strontium ranelate; NA: not available

^a^Tricalcium phosphate (Ca3(PO4)2, TCP), calcium hydrogenphosphate (CaHPO4), calcium carbonate (CaCO3), and hydroxyapatite (HAp)

^b^Equimolar tetracalcium phosphate (Ca4(PO4)2O, TTCP) and anhydrous dicalcium phosphate (CaHPO4, DCPA)

^c^Tetracalcium phosphate (Ca4P2O9, TTCP), dicalcium phosphate dihydrate

Various forms of biomaterials were reported in these studies, including cylindrical, granular/powder, spherical, and disc-shaped. The sites of the bone defects were widely distributed, including the vertebra in one study, humerus in one study, femur and tibia in one study, mandible in two studies, maxilla in two studies, radius in three studies, tibia in three studies, calvaria in four studies, and femur in 18 studies.

### Biomaterial characteristics

The included studies contained multiple types of calcium phosphate materials. Different synthesis methods, crystallinities, particle sizes, implant morphologies, porosities, stoichiometries, and thermal treatments could influence the biological properties and in vivo efficacy of these materials. Table 2 summarizes the characteristics of all materials used in the included research.

**Table 2:** Biomaterials’ characteristics

Study	Biomaterials	Synthesis method	Crystallinity	Particle size	Implant morphology	Porosity	Stoichiometry	Thermal treatment
Baier [22]	CPC^a^/SPC	NA	NA	NA	NA	NA	NA	NA
Carmo [23]	CHA/SrCHA	Precipitation wet method	NA	425- 600 μm	Microspheres	SrCHA presented fewer surface pores than CHA	NA	NA
Cassino [24]	HA/HASr/HAGa	NA	NA	NA	NA	NA	NA	Heated at 1100 °C for 3 h
Chandran [25]	Sham/HA/SrHA	HA powder: wet precipitation method	SrHA did not show any phase change with that of HA	350–400 microns	Micro-granules	SrHA micro-granule majority of pore size: 45–65 µm HA micro-granule: 20–40 µm	HA -Ca/P ratio = 1.67	The dried blocks were biscuit fired at 600 °C to expel the additives and sintered at 1175 °C
Chandran [26]	SrHA—Ca9Sr1(PO4)6(OH)2 /HA–Ca10(PO4)6(OH)2 cHA/cSrHA	SrHA: wet precipitation method	NA	NA	Cylinder	HA: 409 ± 49.39 µm SrHA:265 ± 33.45 µm	NA	Sintered at a high temperature of 1175 °C
Cheng [27]	Sham/CPC^a^/Sr-CPC	NA	NA	NA	Paste	NA	NA	NA
Dagang [28]	HAC/Sr-HAC	NA	NA	NA	Cylinder	NA	NA	NA
Elgali [29]	DBB/HA/SrHA/Sham	HA powde: standardized precipitation method	NA	NA	Granules (GBR Membrane)	NA	HA: Ca/P = 1.67	NA
Ge [13]	PLLA/HA/PLLA/Sr-HA/PLLA	NA	NA	NA	Discs	Sr-HA/PLLA: highly porous and interconnected	Ca/P molar ratio = 1.54	NA
Gu [30]	HA/Sr-HA	NA	NA	NA	Cuboid	HA: Pore size:140 ~ 160 μm Porosity: about 50%	NA	NA
Gu [31]	HA/CPP/SCPP	NA	Sr-doping increased CPP crystal grain size	NA	Cylinder	SCPP, CPP and HA scaffolds possessed interconnected porous network, large pore size (100–400 μm) and an overall porosity of 65%	Ca/Sr molar ratio = 92:8	NA
Guo [32]	nano-HA/SrR nano-HA	Nano-Ha: hydrothermal transformation method	NA	Nano-Ha: irregular in shape with size of 300–450 uM	Granule	NA	NA	NA
Hu [33]	Blank control/HA/Sr-HA	NA	Sr-doping increased HA crystallinity	NA	NA	Both HA and SrHA scaffolds have a porosity of 40%; sr-doping did not affect porosity of HA scaffolds	NA	Temperature was maintained at 1050 °C for 4 min
Kang [34]	Autogenous cancellous bone/SrCPP/CPP	NA	NA	NA	Cylinder	The porosity of all scaffolds is around 86%	Ca/Sr molar ratio = 92:8	NA
Kaygili [35]	HA/SrHA	Sol–gel technique	Crystallite size: 21–27 nm Crystallinity: 69–87%	NA	NA	NA	NA	Calcining at 750 °C for 1.5 h in an electric furnace
Kuang [36]	CPC^b^/Sr-CPC	NA	NA	NA	Cylinder	CPC: 2.15 ± 2.21% 5% Sr-CPC: 1.62 ± 2.42% 10% Sr-CPC: 0.32 ± 1.52%	Sr/(Sr/Ca) molar ratio: 5% and 10%,	NA
Li [14]	HA/SrHA	HA + 10%SrHA: co-precipitation	NA	2 & 5 μm	Rod-shaped	NA	(Ca + Sr)/P = 1.67	Calcined at 1050 °C for 0.5 h
Liao [37]	Blank/HA/Sr-HA	NA	NA	NA	Cuboid	HA: Pore size:140 ~ 160 μm Porosity: about 50%	NA	NA
Luo [38]	3D printed scaffolds: HA/Sr-HA	HA and Sr-HA powders: biomimetic mineralization process HA and Sr-HA scaffolds: 3-D printing	Crystallinity did not seem to change	NA	Discs	Pore size: 800–1000 μm Porosity: HA: 59.3 ± 6.4% Sr-HA: 58.5 ± 3.6%	Sr-HA: (Sr + Ca)/P ratio = 1.58 Sr-HA: Sr/(Sr + Ca) molar ratio = 5.8%	NA
Machado [39]	Blood clots/HA/SrHA	NA	HA with more crystallinity than SrHA	NA	Microspheres	NA	NA	SrHA + HA: Sintered to 1100 °C in a muffle furnace for 27 h
Masaeli [40]	Control/CPC^c^/SrCPC	NA	Sr-doping alters the crystal structure	CPC: 3 mm	Powder	NA	NA	NA
Reitmaier [41]	CPC^a^/SrCPC	NA	NA	NA	Unloaded: Cylinder Loaded: Wedge-shaped	Macroporosity of the printed scaffolds: 50% Pore size: approximately 590 µm	Sr/Ca = 0.123	NA
Salamanna [42]	HA/SrHA/HA-AL7/HA-AL28	Synthesized in N2 atmosphere using 50 ml of solution	Sr-doping reduced the crystals size	NA	Powder	NA	SrHA5: Sr/(Ca + Sr) = 0.05 SrHA10: Sr/(Ca + Sr) = 0.1	NA
Tao [10]	Control/CPC^b^/SrCPC/BSrCPC	NA	NA	NA	Cylinder	NA	NA	NA
Tao [43]	Control/β-TCP/Srβ-TCP/Asp-Sr β-TCP	NA	NA	NA	Cylinder	Sr/β-TCP scaffolds displayed a porosity of 22.1 vol%, the average pore diameter was 1.5 μm	NA	Fired at 1200 °C for 3 h
Thormann [44]	Sham/CPC^a^/SrCPC	NA	NA	NA	NA	NA	Sr/Ca ratio = 0.123	NA
Tian [45]	CPP/SrCPP	NA	The crystal grain size of SCPP was larger	NA	Cylinder	The measured porosity value was about 65% for both scaffolds	NA	NA
Tohidnezhad [46]	Sham/β-TCP/Srβ-TCP	NA	NA	NA	Cylinder	Porosity: β-TCP + Sr Scaffolds: 22.1 vol%. Average pore diameter: 1.5 µm	NA	The filled wax models were fired at 1200 °C for 3 h
Valiense [47]	CHA/SrCHA	NA	NA	425—600 μm	Spheres	NA	NA	NA
Vestermark [48]	HA/SrHA/Allograft	NA	NA	NA	Cylinder	NA	NA	NA
Xie [15]	CPP/SrCPP	Gravity sintering	NA	NA	Cylinder	NA	Ca/Sr = 92/8	NA
Yu [49]	Coll scaffold/(APMs/coll scaffold)/(SrAPMs/coll Scaffold)	SrAPMs: microwave-hydrothermal process	NA	NA	Cylinder	The Coll, APMs/coll and SrAPMs/coll scaffolds were highly porous; pore sizes ranging from 100 to 300 µm	Sr/(Sr + Ca) molar ratio = 0.1	NA
Yuan [17]	HA/SrHA/(HA/G3-K PS)/(SrHA/G3-K PS)	Sol–gel technology	NA	NA	Gel	NA	Ca + Sr/P: 1.5–2	NA
Zarins [11]	Sham/(HA/TCP)/Sr + (HA/TCP)	NA	NA	Sintered ceramic granules: 0.5—1 mm	Granules	Micro porosity and grain size of granules: 400 nm—1 µm	Ca/P and (Ca + Sr)/P molar ratio = 1.67	One to two grams of synthesized calcium phosphate powders were thermally treated at 1100 °C for 1 h
Zhao [16]	WCP/SrWCP/Sr-Ran + WCP	The microwave-assisted H_2_O_2_ foaming method. Hydrothermal treatment	NA	300–450 μm	Cylinder	Highly porous with macropores pore size ~ 100 μm	Sr/(Ca + Sr) molar ratio = 10%	Sintered at 1100 °C for 2 h at a rate of 5 °C/min increment to 1100 °C

CPC: calcium phosphate cement; SPC: Sr-doping calcium phosphate cement; HA: hydroxyapatite; CHA: Carbonated hydroxyapatite; CPP: calcium polyphosphate; TCP: tricalcium phosphate; Sr: Strontium; HAC: hydroxyapatite cement; DBB: Deproteinized bovine bone; Sham: without graft materials; PLLA: poly(l-lactic acid); SCPP: strontium-doped calcium polyphosphate; Asp-Sr/β-TCP: strontium-doped β-tricalcium phosphate (Sr/β-TCP) modified with aspirin; BSrCPC: strontium-doped calcium phosphate cement combined with single-dose local administration of BMP-2;Coll: collagen; APMs: amorphous calcium phosphate porous microspheres; HA/G3-K PS: hydroxyapatite gel modified by integrating branched poly(epsilon-lysine) dendrons with third-generation branches exposing phosphoserine; WCP: hydroxyapatite whiskers; Sr-Ran: strontium ranelate;; NA: not available

^a^Tricalcium phosphate (Ca3(PO4)2, TCP), calcium hydrogenphosphate (CaHPO4), calcium carbonate (CaCO3), and hydroxyapatite (HAp)

^b^Equimolar tetracalcium phosphate (Ca4(PO4)2O, TTCP) and anhydrous dicalcium phosphate (CaHPO4, DCPA)

^c^Tetracalcium phosphate (Ca4P2O9, TTCP), dicalcium phosphate dihydrate

### Risk of bias and quality assessment

**Fig. 3:**
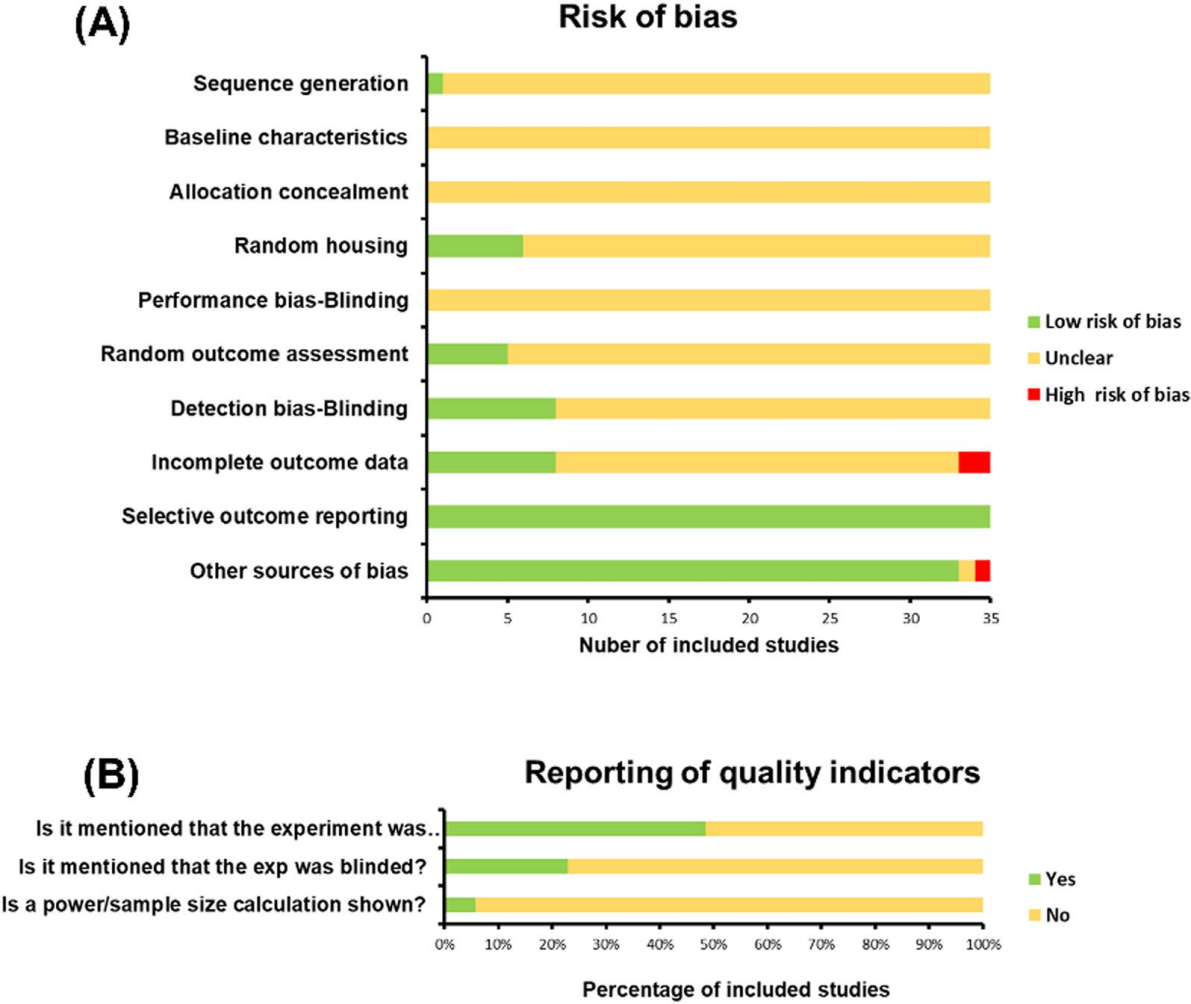
Risk of bias (Graph **A** displays the risk of bias in all included studies which were assessed using SYRCLE's bias risk tool. Graph **B** displays the reporting of three key quality indicators)

The risk of bias of the included studies was relatively high (Fig. 3A). Among them, only one paper [29] provided a sufficient and reasonable description of the generation of random sequences. Furthermore, it was difficult to confirm the accurate baseline characteristics in each group as none of the studies offered complete baseline information. None of the papers reported on the ‘allocation concealment’ and ‘blinding of performance bias’. ‘Random housing’ was considered as a ‘low risk of bias’ in six publications [10, 24, 33, 42, 43, 46] (17%), and only five of the articles [30, 31, 34, 37, 48] (14%) reported ‘random selection for outcome assessment’. Eight articles [15, 23, 26, 34, 39, 44, 47, 48] (23%) were considered to have a ‘low risk of bias’ in terms of the ‘blinded outcome reviewers’, while two papers [22, 44] were considered to have a ‘high risk of bias’ in terms of ‘incomplete data reporting’. Moreover, in terms of ‘selective outcome reporting’ and ‘other sources of bias’, a majority of the articles were defined as having a ‘low risk of bias’.

Another three quality indicators for the 35 studies are presented in Fig. 3B. For 17 studies (less than 50%), it was reported that the experimental groups were randomized in some way. Less than 1/4 of the studies reported ‘blinding of the experiment’, and only two articles [23, 48] mentioned the ‘power/sample size calculation’.

### Meta-analysis of new bone formation from histological outcomes

**Fig. 4:**
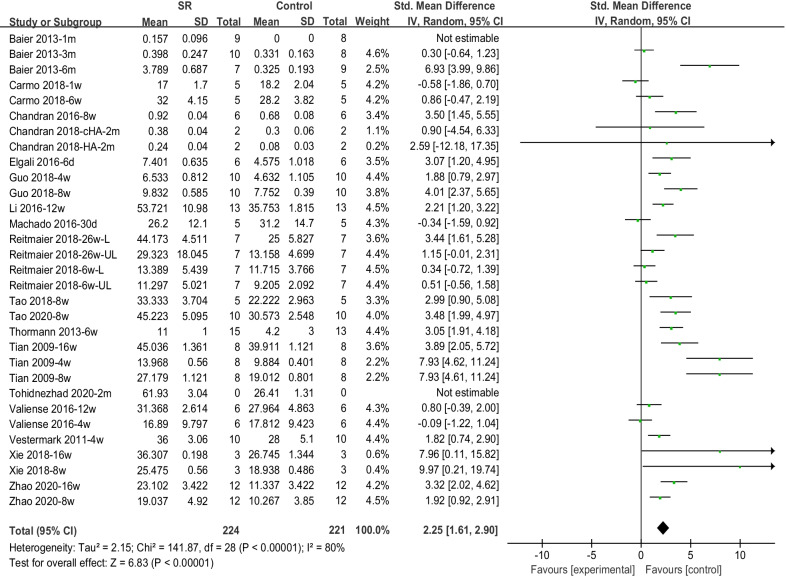
Forest plot of NBF of histological outcomes

A total of 18 articles were included in our meta-analysis, covering 31 comparisons and 445 defects. In this analysis, the pooled effect for NBF was 2.25 (95% CI 1.61–2.90), indicating a significantly higher NBF for Sr-doped materials (Fig. 4).

Table 3 shows the outcomes of the subgroup analysis for NBF. In both the healthy animal group and osteoporosis models, the Sr-enriched material significantly increased NBF, with (SMD: 1.85 [0.95, 2.76], I^2^ = 81%) and (SMD: 2.73 [1.94, 3.52], I^2^ = 71%), respectively. According to the included studies, a superior bone repairing effect could be found in healthy animals. A forest plot of this is provided in Additional file 3: Fig. S1. For animal models, one rat study [23], one rabbit study [47], and one sheep study [39] reported lower NBF in the Sr-doped group without statistical significance during short implantation periods (1 week–30 days). Results of other studies and meta-analyses all suggested that Sr doping could significantly promote NBF (Additional file 3: Fig. S2). Sub-group analyses of different calcium phosphates (HA, β-TCP, CPC, and CPP) and different follow-up periods (1 month, 2 months, 3 months, and 4 months) both supported the conclusion that Sr-doping enhanced NBF. However, high heterogeneity could be observed in all subgroups, with I^2^ values ranging from 52 to 87% (Additional file 3: Fig. S3 and Additional file 3: Fig. S4).

**Table 3:** Subgroup analysis of the included papers for outcome new bone formation (NBF; SMD)

Subgroup	Number of comparisons	Number of defects	Effect estimate SMD [95% CI]	Heterogeneity (I^2^)
**Disease**				
Health	17	202	1.85 [0.95, 2.76]	81%
Osteoporosis	14	243	2.73[1.94, 3.52]	71%
**Animal**				
Rat	15	267	2.42 [1.62, 3.22]	79%
Rabbit	7	84	4.32 [1.78, 6.86]	88%
Sheep	7	74	0.85 [− 0.03, 1.72]	52%
**Material**				
HA	7	120	2.18 [1.19, 3.17]	74%
β-TCP	2	20	3.48 [1.99, 4.97]	Not applicable
CPC	9	145	1.98 [0.85, 3.12]	83%
CPP	5	60	6.60 [4.09, 9.12]	52%
**Period**				
1 m	6	95	1.68 [0.08, 3.29]	86%
2 m	10	116	3.52 [2.35, 4.69]	55%
3 m	2	44	1.10 [− 0.08, 2.29]	74%
4 m	3	46	3.59 [2.54, 4.64]	0%

HA: hydroxyapatite; β-TCP: beta-tricalcium phosphate; CPC: Calcium phosphate cements; CPP: Calcium polyphosphate; m: month

### Meta-analysis of new bone formation from micro-CT assessment

**Fig. 5:**
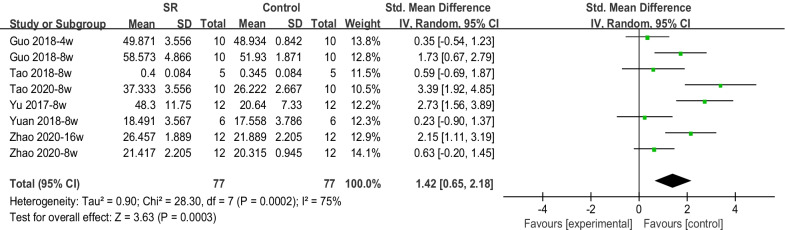
Forest plot of BV/TV

Micro-CT measurements of bone volume/tissue volume (BV/TV) were performed in five of the articles included in the meta-analysis. The overall effect of BV/TV was 1.42 (95% CI 0.65–2.18, p < 0.05), suggesting that Sr enrichment promoted NBF and bone regeneration (Fig. 5).

### Meta-analysis of the remaining materials

**Fig. 6:**
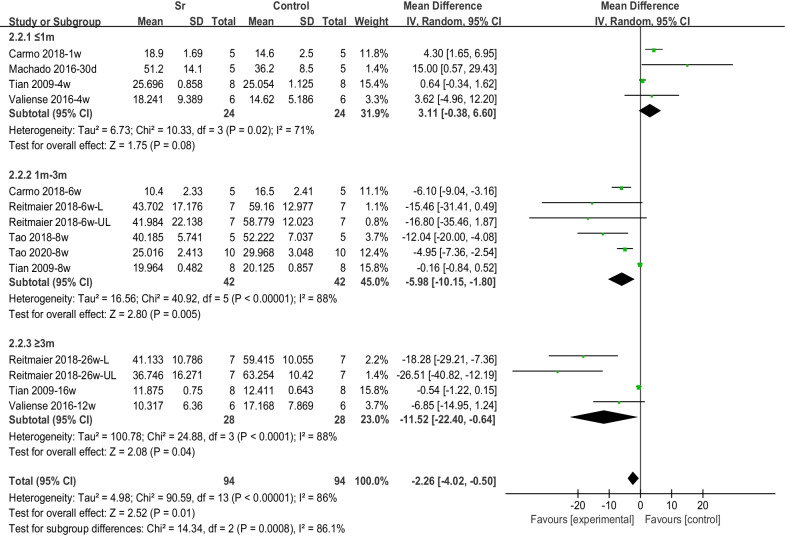
Forest plot of RM-subgroup analysis by period

In terms of material absorption, the histological outcomes were extracted from six articles, among which four comparisons found that material remained for less than 1 month, six comparisons between 1 and 3 months, and four comparisons for more than 3 months. The results showed that, in the early stages (≤ 1 month), the absorption of the Sr-doped material was less than that of the non-Sr-doped group (3.11 [− 0.38, 6.60]). In the middle (1–3 months) and longer (> 3 months) periods, the absorption of the Sr-doped material was significantly higher than that of the Sr-free group (Fig. 6).

**Fig. 7:**
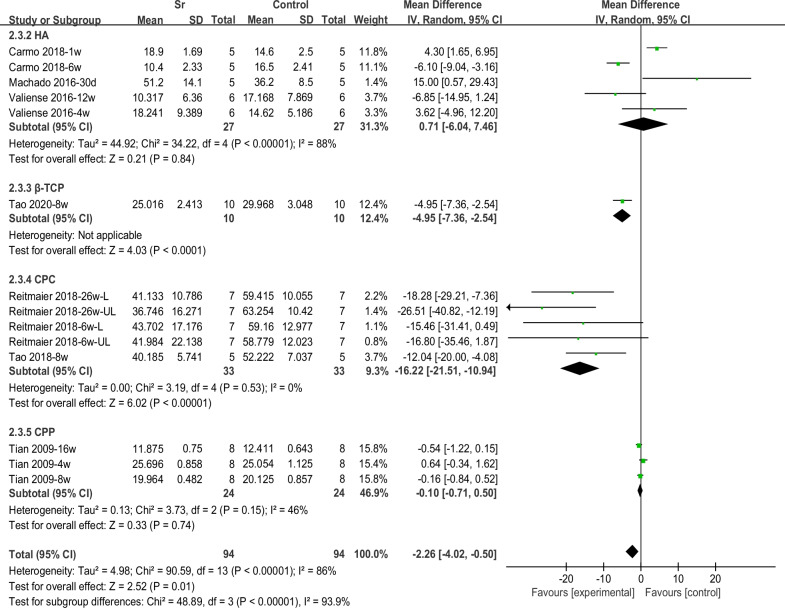
Forest plot of RM-subgroup analysis by materials

Subgroup analysis was also conducted for different material types (HA, β-TCP, CPC, and CPP). The results showed that the absorption of Sr-doped HA materials was slower than that of Sr-free materials, albeit with no statistical significance. For the other three types of materials, the absorption of Sr-doped materials was faster than that of the control group. The differences between β-TCP and CPC were statistically significant (Fig. 7).

### Publication bias

Funnel plots showed no significant publication bias, as no significant asymmetry was detected (Additional file 3: Fig. S5).

## Discussion

Synthetic calcium phosphate bone substitutes have been widely used for bone defect regeneration. To overcome the limitations of calcium phosphate materials, researchers are continuously proposing new methods. In recent years, many researchers have focused on adding inorganic ion Sr to calcium phosphate materials to improve their in vivo performance. However, at present, there is no consensus on whether Sr supplementation can significantly promote the biological and in vivo efficacy of bone replacement materials, to the best of our knowledge. Therefore, this paper systematically reviewed relevant in vivo studies and conducted a quantitative meta-analysis. The results showed that the Sr-enhanced material significantly promoted the formation of new bone in the bone defect area, and the material was more easily absorbed. This is similar to the results of a previous study [50].

### Bone formation

The specific mechanism by which Sr-containing materials promote osteogenesis is still unclear. Bone morphogenetic protein-2 (BMP-2)/Smad-1 and the osteoprotegerin (OPG)/receptor activator of the nuclear factor‑κB ligand (RANKL) are two important signalling pathways for regulating osteogenesis. Previous studies have shown that bone remodelling regulates osteoblasts and osteoclasts through the BMP-2/Smad1 and OPG/RANKL signalling pathways, and is capable of bi-directional signalling [51, 52]. Sr is believed to have both osteogenic (anabolic) and antiabsorptive (catabolic) effects [36, 53]. Many studies have shown that the addition of Sr could stimulate the differentiation of MSCs or other osteoblast lineages [54, 55]. The expression of osteoblast markers (alkaline phosphatase [ALP], bone sialoprotein, and osteocalcin) was increased to promote the formation of bone nodules [53, 56], while reducing the differentiation and proliferation of osteoclasts [57].

Osteoporosis is a systemic bone disease characterized by bone loss and structural destruction. Owing to osteoblastic degeneration, increased osteoclast function, and insufficient bone formation ability, the treatment of bone defects in patients with osteoporosis is very challenging [1] In this study, a meta-analysis of nine studies [10, 14, 16, 22, 25, 26, 32, 43, 44] using osteoporosis models was conducted, and the results showed that the addition of Sr could significantly promote NBF in animals with osteoporosis.

Sr has been shown to promote NBF by activating CA-sensitive receptors and inhibit bone resorption by blocking the expression of receptor activators of the nuclear factor κB ligand (RANKL) [58, 59]. Animal studies on Sr-doped materials have shown that the enhancement of bone formation could be related to the release of Sr ions during the degradation process [45]. Biomaterials containing Sr exhibit high expression of physiologically active signalling molecules, such as OPG, NFkB 105, ALP, Col-1α, osteocalcin, osteopontin, and BMP 2/4 [57, 60–63]. This means that Sr-rich materials stimulated the release of these molecules more than calcium phosphate alone or simply the trauma itself.

In addition, the Sr released by bioceramics has been shown to stimulate angiogenesis by increasing the secretion of the cytokines that promote cell angiogenesis [64, 65]. A previous study has shown that, one week after SrWCP implantation in osteoporotic animals, vascular-like structures were formed in the pores in the central region of the bioceramics [16]. This angiogenesis is necessary for bone regeneration because these new blood vessels supply the oxygen, nutrients, and cells required for bone formation.

In addition, different animal models, implant sites, and bone defect sizes may also influence the conditions of NBF and material degradation. It is generally believed that experimental research on large animals reflects clinical practice more closely; however, there are few studies using large animals. Only three studies on sheep have been included in this meta-analysis on NBF, while no meta-analysis on remaining material could be conducted owing to the limited number of studies on large animals. The subgroup analysis of different animal types showed that Sr-doping significantly promoted the formation of new bone in sheep, dogs, rabbits, and rat. However, it should be noted that, although subgroup analyses were conducted, the results of these meta-analyses still exhibit significant heterogeneity among studies. This could be related to differences in implant sites (calvaria, femur, radius, etc.), bone defect sizes (3 mm, 5 mm, 10 mm, etc.), sample size, and experimental design.

### Material degradation

Histological assessments were used to quantitatively determine the residual materials by conducting a meta-analysis. The percentages of remaining materials according to different implantation periods are shown in Fig. 6. At less than 1 month, the degradation rate of Sr-doped materials was lower than that of the control group. However, the degradation rate of the Sr-doped group was significantly higher at longer periods (greater than 1 month). This indicates that the degradation rate of Sr-doped materials may gradually increase with time, and is significantly higher than that of the Sr-free group. Studies showed that different types of calcium phosphate would affect the degradation rate of materials. It is generally believed that HA is more difficult to degrade. In the subgroup analysis for different material types, it can be seen that the residual rate of Sr-doped materials in the HA group is higher than that in the control group. However, the three studies [23, 39, 47] in the HA group with high material residual rates all had shorter observation periods (1 w, 4 w, and 30 d). Therefore, this may suggest that the doping of Sr has a time-dependent effect on the material absorption.

Although enhanced degradations of Sr-doped materials were reported in studies in vitro and in vivo, the underlying mechanism remains unclear and requires further investigation. Some researchers believe that the degradation rate of CPP scaffolds in vivo is usually affected by the initial size of the particles during scaffold formation, the scaffold structure, the implantation site, and other factors [66]. The doping of Sr was generally carried out through ion substitution, where Sr^2+^ could replace Ca^2+^ ions. Previous studies have shown that the ion radii of bioinorganic ions usually differ from those of substituted ions, and their supplementation could change the crystallinity, lattice parameters, crystal size, morphology, stability, biological activity, bone conductivity, and solubility of the material [6, 13, 40]. These physical and chemical changes may alter the fragmentation and biological absorption of biomaterials [13, 47]. According to Chandran et al. [25] and Landi et al. [67], the increased dissolution rates could be a result of the combined action of the increased pore size and the amorphous properties of SrHA particles.

In our opinion, the faster degradation rates of Sr-doped materials could also contribute to the improved release of bioinorganic substances and, thus, accelerate NBF.

### Implications and limitations

Our study is likely to be the first report that systematically reviews relevant studies on Sr-doped (CaP)-based materials and conducts sub-group meta-analyses according to different influence factors. Furthermore, our study revealed the effect of Sr-enhanced materials in vivo, which provides a good basis for their further research and clinical application.

However, our study also has certain limitations. First, in this study, high heterogeneity was found in the meta-analysis of NBF and residual materials. Subgroup analyses based on material type, implantation period, experimental animal species, etc., also had high heterogeneity. In view of the significant heterogeneity among the studies included in our meta-analyses, caution should be exercised when generalizing our conclusions. It is suggested that homogenized study settings should be adopted in subsequent studies to provide more convincing evidence for clinical applications. Second, the quality of the included studies is not high enough. The details of sample size estimation and randomization methodology were not found in most studies. Finally, although Sr has a beneficial effect on bone formation, its potential negative effects should also be taken into account, especially in high doses [29, 68, 69]. A dose-dependent effect of Sr on osteoblasts could be detected in some in vitro studies [70]. Animal studies have shown that the Sr dosage was very important, as high doses could cause osteomalacia [71]. In this study, the included studies used different concentrations of Sr, and some did not report relevant data. Therefore, it is necessary to further explore the optimal concentration of Sr.

### Relevant studies during 2021

During the past year (2021), another four in vivo studies relevant to this topic were found. One of them focused on  strontium-doped nano hydroxyapatite-gelatin (Sr-nHA-Gel). An in vitro study and the in vivo repair of critical-sized cranial defects confirmed that Sr-nHA gel had relatively effective bone regeneration ability [72]. Another article focused on strontium-releasing nanoscale cement. In vivo and in vitro experiments showed that SR nano bone cement had the dual effects of osteoclast inhibition and osteogenic stimulation, indicating good potential for the treatment of osteoporotic bone defects [73]. The effect of the scaffold degradation rate on osteogenesis has been widely researched. Miao et al. [74] prepared strontium-doped calcium sulfate (SrCSH) and strontium-doped tricalcium phosphate microsphere (Sr-TCP) scaffolds. In the experiment on repairing osteoporotic femoral defects, they found that, when the degradation rate of the scaffold matched the growth rate of new bone, the rapid repair of osteoporotic bone defects was promoted. In contrast, the slow degradation of scaffold materials hindered the growth of new bone to a certain extent. This study further clarified the importance of the scaffold degradation rate in the repair of osteoporotic bone defects. Vascularized bone tissue engineering is of great significance for the reconstruction of critical bone defects. The application of calcium phosphate cement in vascularized bone tissue engineering is limited due to the lack of consequent angiogenesis and unsatisfactory physical and chemical properties. Wu et al. [75] developed a strontium-reinforced calcium phosphate composite cement based on the reported osteogenic and angiogenic properties of CPHC-star and BaSO4-incorporated calcium phosphate hybrid cement; further, Sr ions could improve the biological and physicochemical properties of CPC. In vivo and in vitro studies have shown that the material has the dual potential of osteogenesis and angiogenesis.

The aforementioned studies exhibited the significance of strontium-doped bone substitute materials in promoting bone regeneration, and also formed the basis for research into bone substitute materials.

## Conclusion

According to the results of the systematic review and meta-analyses herein, Sr supplementation is advantageous in terms of promoting NBF and accelerating material degradation. The type of material (HA, β-TCP, CPC, or CPP) does not seem to affect NBF. In terms of material degradation, HA seems to degrade slowly, while the other three categories degraded more rapidly. However, the existing meta-analysis results all suggested high heterogeneity and no statistical significance. Therefore, further research is required to verify the differences between materials and further verify the conclusions of this study. Determining the optimum concentrations of Sr and the best Sr-doped calcium phosphate materials is an important future research direction. In addition, the angiogenic potential of materials could be another research direction worth focusing on, in addition to osteogenesis.

## Supplementary Information

**Additional file 1: Table S1.** Literature search-strategy for PubMed.

**Additional file 2: Table S2.** Literature search-strategy for Embase (via OVIDSP).

**Additional file 3:** Supplementary figures.

## Data Availability

Data analysed during this study was included in this article and its supplementary information files.

## Abbreviations

Sr: Strontium
NBF: New bone formation
RM: Remaining material
BV/TV: Bone volume/tissue volume
SYRCLE: Systematic Review Centre for Laboratory Animal Experimentation
RoB: Risk of Bias
95% CI: 95% Confidence interval
SMD: Standardized mean differences
MD: Mean differences
CPC: Calcium phosphate cement
SPC: Sr-doping calcium phosphate cement
HA: Hydroxyapatite
TCP: Tricalcium phosphate
TTCP: Anhydrous dicalcium phosphate
DCPA: Tetracalcium phosphate
CHA: Carbonated hydroxyapatite
CPP: Calcium polyphosphate
HAC: Hydroxyapatite cement
DBB: Deproteinized bovine bone
PLLA: Poly(l-lactic acid)
SCPP: Strontium-doped calcium polyphosphate
Asp-Sr/β-TCP: Strontium-doped β-tricalcium phosphate (Sr/β-TCP) modified with aspirin
BSrCPC: Strontium-doped calcium phosphate cement combined with single-dose local administration of BMP-2
Coll: Collagen
APMs: Amorphous calcium phosphate porous microspheres
HA/G3-K PS: Hydroxyapatite gel modified by integrating branched poly(epsilon-lysine) dendrons with third-generation branches exposing phosphoserine
WCP: Hydroxyapatite whiskers
Sr-Ran: Strontium ranelate
NA: Not available
ALP: Alkaline phosphatase
BMP-2: Bone morphogenetic protein-2
OPG: Osteoprotegerin
RANKL: Receptor activator of the nuclear factor‑κB ligand
Sr-nHA-Gel: Strontium-doped nano hydroxyapatite-gelatin
