# Supplementary material for: Does the incorporation of strontium into calcium phosphate improve bone repair? A meta-analysis
Source: BMC Oral Health. 2022 Mar 8;22:62. doi: 10.1186/s12903-022-02092-7 (PMC8905839; doi:10.1186/s12903-022-02092-7)
Supplement: Supplementary file 3 — Additional file 3: Supplementary figures. [file 12903_2022_2092_MOESM3_ESM.docx]

**Figure S1.** Forest plot of NBF-subgroup analysis by health condition

**Figure S2.** Forest plot of NBF-subgroup analysis by animal

**Figure S3.** Forest plot of NBF-subgroup analysis by material

**Figure S4.** Forest plot of NBF-subgroup analysis by implantation periods


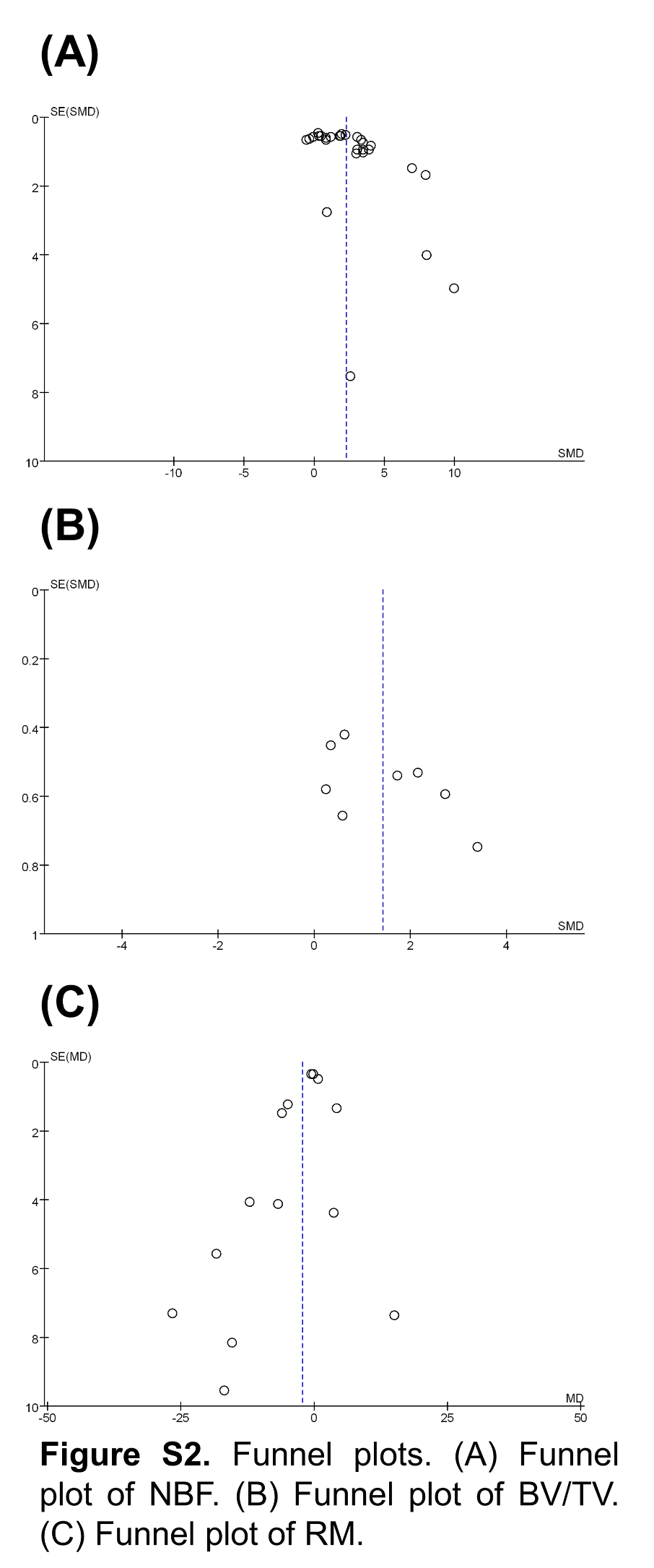


**Figure S5.** Funnel plots. (A) Funnel plot of NBF. (B) Funnel plot of BV/TV. (C) Funnel plot of RM.
